# Metastatic Sarcomatoid Renal Cell Carcinoma: A Journey From the Kidney to the Gingiva

**DOI:** 10.7759/cureus.63827

**Published:** 2024-07-04

**Authors:** Zainab Kamal, Chandergupt Singh, Shruti Tandon, Arundeep K Lamba, Aadithya B Urs, Shivangni Rajoria

**Affiliations:** 1 Periodontics, Maulana Azad Institute of Dental Sciences, New Delhi, IND; 2 Oral Pathology and Microbiology, Maulana Azad Institute of Dental Sciences, New Delhi, IND

**Keywords:** immunohistochemistry, mandibular gingiva, sarcomatoid, metastasis, renal cell carcinoma

## Abstract

Oral metastatic lesions are very rare and are often diagnosed at a later stage, complicating treatment. Renal cell carcinoma (RCC) is the third most frequent neoplasm to metastasize to the oral cavity, following breast and lung cancers. These metastatic lesions are usually asynchronous and develop after the initial diagnosis, affecting the overall survival rate. This report describes a known case of RCC with a growth in the mandibular gingiva. Multiple pulmonary and femoral metastases appeared five years after the initial renal lesion. The gingival growth was excised and referred for histopathological examination, which revealed a pleomorphic sarcomatoid cellular morphology. Immunohistochemistry with an array of markers led to the diagnosis of sarcomatoid RCC, a rare high-grade tumor. This case underscores the importance of detailed history-taking, interpretation of clinical findings, and emphasis on histopathological examination to arrive at a conclusive diagnosis.

## Introduction

Distant metastasis to the oral cavity is very rare and accounts for 1% of all oral malignancies [1]. Renal cell carcinoma (RCC) is the third most frequent neoplasm that metastasizes to the oral cavity, following breast and lung cancers [2]. Metastases from RCC generally involve the lungs, regional lymph nodes, liver, bones, and brain. However, metastases to the oral cavity are extremely rare, accounting for only 6%, with the tongue being predominantly involved, followed by the gingiva and maxillary bones [3]. Metachronous RCC metastasis is very uncommon and can be discernible during follow-up even years after radical nephrectomy. Widespread metastatic lesions cannot be surgically cured, and systemic therapy is required [4]. Furthermore, sarcomatoid differentiation in the metastatic lesion, compared to its primary histological type, is particularly severe. Twenty percent of patients with metastatic RCC undergo sarcomatoid dedifferentiation, which not only impairs the prognosis but also leads to a dismal survival rate [5]. This paper describes the clinical and pathological aspects of another oral cavity metastatic RCC patient with aggressive sarcomatoid differentiation.

## Case presentation

A 57-year-old non-smoker male presented to the Department of Periodontics, Maulana Azad Institute of Dental Sciences, New Delhi, in July 2022 for examination of a right mandibular gingival growth that had been present for four months. Eight years earlier, he had undergone radical nephrectomy with retroperitoneal lymph node dissection for right kidney RCC. After surgery, the patient was on regular follow-up with blood investigations and full body positron emission tomography (PET) scans. Five years following the surgery, a full-body PET scan revealed several lung and femur metastases of RCC, although no fludeoxyglucose F18 (FDG) uptake was seen in the jaw region. Since then, he had been on multiple immunochemotherapeutic medications such as Tab. Cabozantinib, Inj. Pembrolizumab, Tab. Lenvatinib, and Inj. Zoledronic acid.

**Figure 1:**
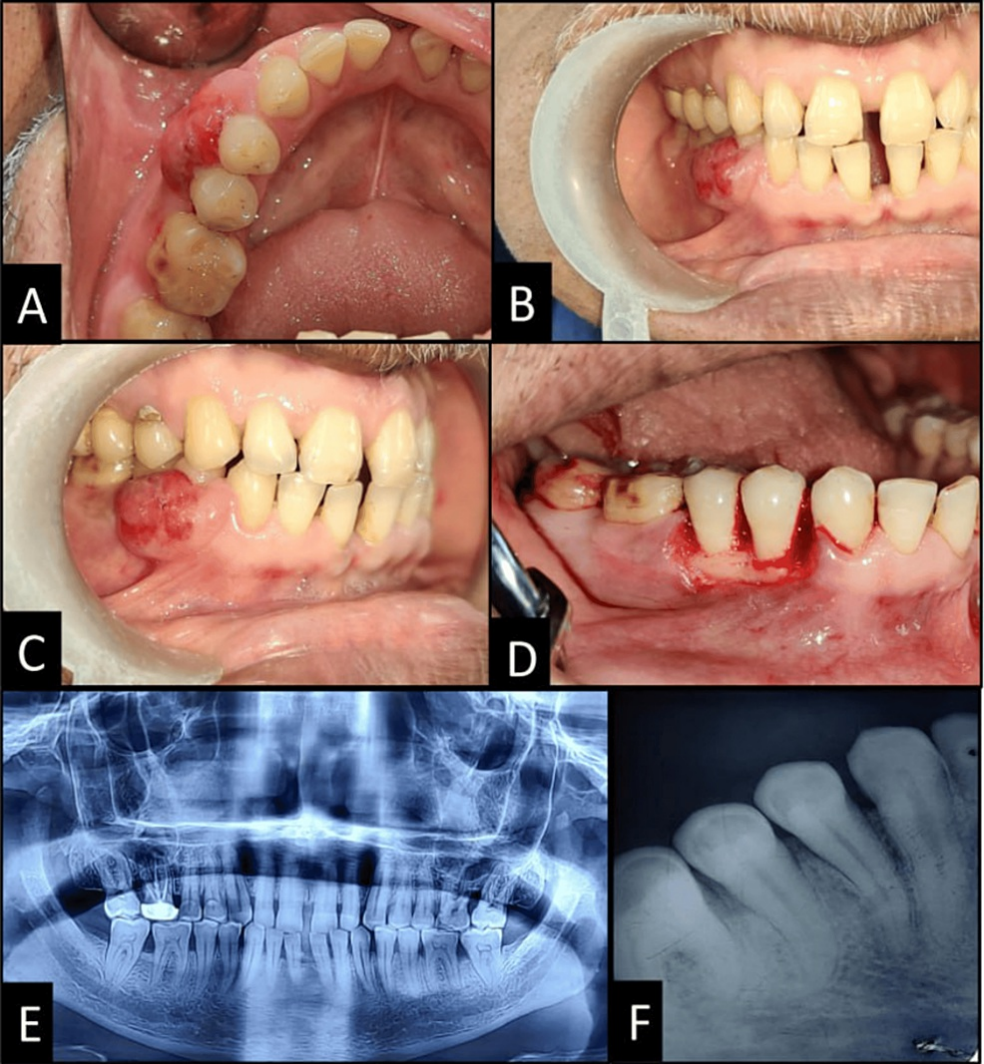
Clinical photograph showing: (A, B, C) Gingival overgrowth present in the mandibular right premolar region; (D) Complete excision of the growth and debridement; (E) Preoperative orthopantomogram (OPG); (F) Preoperative intraoral periapical radiograph (IOPAR) in relation to 44, 45.

On clinical examination, a localized gingival growth was present on the buccal surface, extending from the distal surface of the mandibular right canine to the distal surface of the second premolar, involving the marginal, papillary, and attached gingiva, measuring approximately 15mm x 10mm. The growth was a slow-growing, pinkish-red, sessile mass which was soft, non-tender, and not fixed to the underlying bone on palpation (Figures 1A-1C). The false probing depth in the region was 5mm. No palpable cervical lymphadenopathy was present. Panoramic and intraoral periapical radiography revealed periodontal space widening in relation to 44 and 45 and crestal bone loss in relation to 44, 45, and 46. There was slight radiolucency at the furcation area of 46, suggesting trauma from occlusion (Figures 1E-1F). Based on the clinical aspects, benign peripheral lesions were considered as a provisional diagnosis, and a biopsy was further planned after medical oncology clearance.

All hematological and biochemical parameters were within the normal range. No preoperative medication was prescribed. An excisional biopsy was performed for the histopathological analysis of the tissue after obtaining informed consent from the patient under local anesthesia (lignocaine 2% with 1 in 80,000 adrenaline). At the apical border of the growth, an external bevel incision was made using a #15 scalpel blade, extending occlusally from the distal surface of the canine to the distal surface of the second premolar. This was followed by a crevicular incision, and the lesion was separated from the alveolar bone with a periosteal elevator. The area was thoroughly debrided and irrigated with normal saline (Figure 1D). The flap was undermined from the sides, and a single sling suture was placed in relation to 44, 45 with 3-0 silk suture. The patient was advised to take Amoxicillin 500mg three times a day (TDS) and Diclofenac 50mg twice a day (BD) for five days and was recalled after one week for suture removal. A liquid or soft diet was recommended for the first 24 hours, and the patient was advised to avoid eating hard, crunchy, or spicy foods.

The biopsied tissue was placed in a leak-proof container with 30 mL of 10% formalin. The container was labeled with the patient's details and sent to the laboratory in a plastic bag for microscopic analysis of the tissue.

Histology

**Figure 2:**
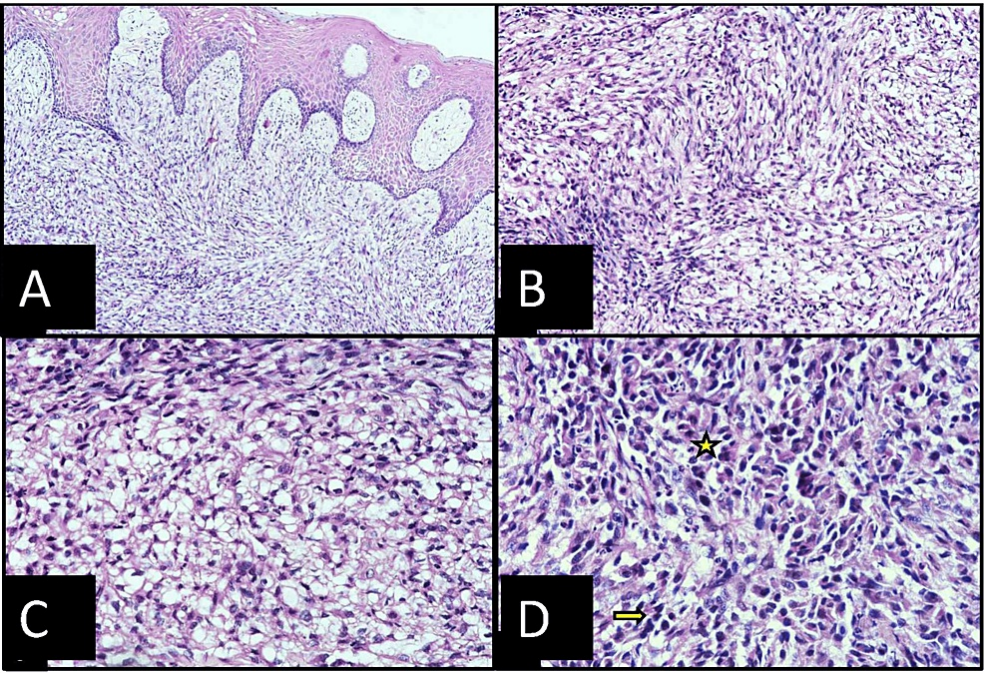
Histopathological features showing: (A) tumor cells abutting the epithelium (10x), (B) alternating areas of epithelioid and clear cells (20x), (C) clear vacuolated cells with peripherally pushed nuclei (40x), and (D) sarcomatoid cells exhibiting atypical features such as hyperchromasia (arrow) and mitosis (asterisk) (40x).

Microscopic examination of H&E stained slides revealed that the lesional tissue was composed of clear and spindle-shaped cells in a moderately collagenous background covered with parakeratinized stratified squamous epithelium. Cuboidal clear cells with peripherally placed nuclei were observed in some areas, alternated with polygonal epithelioid cells with eosinophilic cytoplasm and hyperchromatic nuclei. The spindle-shaped cells showed sarcomatoid characteristics, including hyperchromasia, and cellular and nuclear pleomorphism. A few mitoses were found in both polygonal and spindle cells (Figure 2). Foci of granular eosinophilic necrotic material were seen. Differential diagnoses of clear cell lesions of the oral cavity included clear cell odontogenic carcinoma, and because of the sarcomatoid appearance, leiomyosarcoma, synovial sarcoma, and metastatic RCC were considered. In the routine panel of immunohistochemical analysis, pancytokeratin and vimentin were diffusely positive while positive epithelial membrane antigen (EMA) expression was focally noted in epithelioid polygonal cells. Moderately intense nuclear positivity was noted with PAX8, while S100 and smooth muscle actin immunomarkers were negative. Correlating with clinical history and histopathological findings, a definitive diagnosis of metastatic sarcomatoid RCC was finalized.

Following the histopathological diagnosis, the patient was referred to an oncology center for further comprehensive treatment.

## Discussion

Metastasis of RCC is rather prevalent, occurring in 40-50% of cases [2]. In the head and neck region, it metastasizes in 6% of cases, predominantly to the thyroid, parotid glands, and sinus [3]. Oral metastases are reported in patients between the ages of 1 and 87 years, with a mean age of 58.2 years. Males are adversely impacted, with a male to female ratio of 3.1:1. The most common sites involved are the tongue followed by the mandible, gingiva, maxilla, lip, soft palate, and buccal mucosa [6]. In our case, the affected male patient was in his fifties with multiple metastases including a gingival lesion.

Distant metastasis from the primary tumor most commonly occurs in the lungs and bones, chiefly through the hematogenous route [3]. The valveless Batson venous plexus is a culprit for retrograde venous flow which facilitates the spread to the head and neck region [6]. Another possible theory involves retrograde lymphatic channels, through which the flow reaching the subglottis bypasses the intercostal, mediastinal, or supraclavicular lymph vessels, leading to seeding of tumor cells in the head and neck region [7].

Metachronous lesions are found to have a better prognostic outcome than synchronous lesions [4]. Around 50% of oral lesions are represented as the initial clinical manifestation of the primary underlying RCC, which indicates a poor prognosis [8]. In the present case, metastases were found five years after the surgical removal of the kidney. Metachronous metastases are identifiable in routine follow-up through advanced techniques like CT scan, MRI, or PET scans [8]. In this case, the most recent PET scan showed multiple FDG-avid skeletal lesions and bilateral lung nodules, although no aberrant FDG uptake was noted in the jaw region. This may be due to the fact that the gingival lesion is superficial with no bone involvement and might be diagnosed at a very initial stage.

On clinical examination, the lesion on the mandibular gingiva appeared benign. No abnormalities were detected on intra-oral and panoramic radiographs. All features were directed towards the differential diagnosis of a benign lesion except for the history of RCC treatment.

Oral metastases of RCC are uncommon; only a handful of cases are documented in the literature. In a review article by Nisi M et al., 132 cases of oral metastasis of RCC were reported from 1911 to 2020 [6]. Among the reported cases, the tongue was the most common site for renal metastasis (40.91%), with metastasis to gingiva only in 16.66% of cases. After a thorough literature search of reported cases of metastasis of RCC to the oral cavity after the year 2020, a total of 20 articles were found. Among them, cases with metastasis to the mandible were more frequent (five cases), followed by the tongue and buccal mucosa (four cases each). Only three cases of metastasis to the gingiva were reported. Oral metastases were more frequent in elderly males. In a few cases (seven cases), the oral lesion was the first presentation, and the primary lesion in the kidney was discovered later, which marks a late stage complicating the treatment. Complete excision of the lesion with wide margins should be performed to prevent recurrence. Metastasis involving the mandible and tongue is treated by mandibulectomy and glossectomy, respectively. Metastasis to distant organs affects the mean survival rate of the patient, and palliative care is needed.

The clinical presentation can easily masquerade as oral benign lesions such as pyogenic granuloma, peripheral giant cell granuloma, or fibrous epulis. In this case, the lesion showed no evidence of bone abnormality with a slow-growing course, which led to the differential diagnosis of a benign lesion, except for the history of RCC treatment. Histological examination is required for the confirmation of the diagnosis for the treatment course.

**Figure 3:**
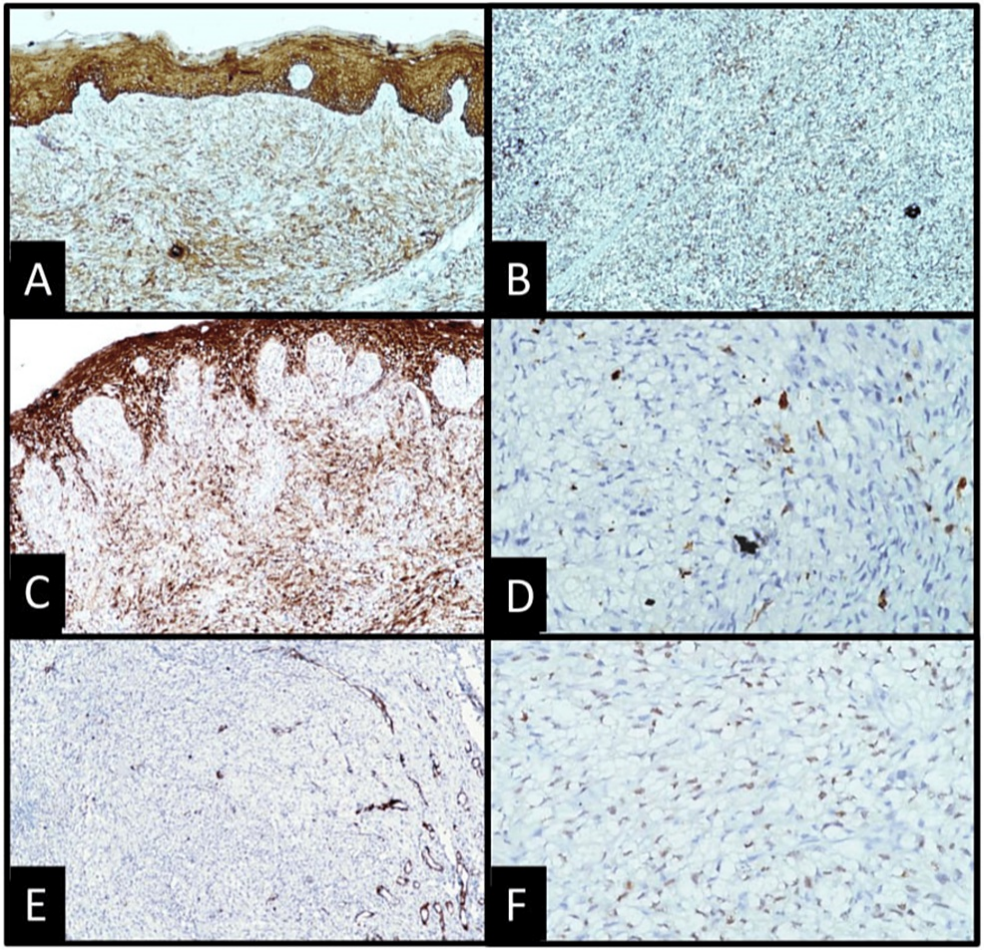
Immunohistochemical reactivity in tumor cells is positive for (A) Cytokeratin AE1/3, 10x; (B) Vimentin, 10x; and (C) Epithelial Membrane Antigen, 10x. Immunohistochemically, lesional tissue is negative for (D) S-100, 40x and E: Smooth Muscle Actin, 10x. F: Nuclear immunopositivity is seen in carcinomatous cells for PAX 8 (40x).

Among the several histologic types of RCC, clear cell RCC is the most common, accounting for 75-80% of all cases. Sarcomatoid differentiation, however, has been noted in only 10% of all RCCs [9]. This differentiation is related to aggressive tumor behavior, reflecting its poor prognosis. In metastatic RCC, approximately 20% exhibit sarcomatoid differentiation [5]. According to a review by Nisi M et al., metastatic RCC gingival lesions are found in only 16% of metastatic RCC oral cavity lesions [6]. To the best of our knowledge, this is the first case presentation of metastatic RCC with sarcomatoid differentiation in the gingiva. In this case, due to the different cellular morphologies, we faced difficulty arriving at a final diagnosis. Although the primary lesion in the renal region was diagnosed as a clear cell variant of RCC, with the distant metastasis, the tumor showed higher-grade changes toward dedifferentiation. Two cell populations were noted: the epithelioid cells showed more cohesiveness and less cellular atypia with well-maintained cellular architecture, while sarcomatoid components were less cohesive with a high nuclear grade and changes in cellular shape and architecture. The clear cell morphology and arrangement were found to be altered with the loss of normal organoid architecture and showed extensive dedifferentiation towards mesenchymal changes, which is very uncommon and inconsistent with conventional clear cell RCCs seen in the literature [1]. The existence of these clear cells could be confused with the clear cell variant of mucoepidermoid carcinoma, which was ruled out by periodic acid-Schiff (PAS) staining showing negativity for clear cells [10]. A few cells showed elongated nuclei with typical “cigar-shaped” nuclei, and the differential diagnosis of leiomyosarcoma was considered, but immunohistochemistry found S-100 to be negative. With an array of immunohistochemical markers, the final diagnosis of RCC was confirmed by positive pancytokeratin, vimentin, and RCC-specific PAX8 antigen (Figure 3). These immune results are consistent with those reported by Vasilyeva D et al., Kudva R et al., and Georgy JT et al. [11-14]. PAX8 has been found to be a useful marker for metastatic RCC.

The sarcomatoid differentiation in the epithelial cells can be explained by the epithelial-mesenchymal transition due to which the mesenchymal cellular changes in the epithelial cells are responsible for the distant metastasis of the tumour cells [5]. RCC with sarcomatoid differentiation worsens the prognosis and has an unfavorable overall survival. It is found that this differentiation responds poorly to systemic therapy [15].

The primary treatment modality for primary and metastatic RCC is surgical excision as a standard procedure, but with the increased understanding of molecular biology, target-based therapeutic strategies are now commonly used. The cellular biology is different in the sarcomatoid type than in conventional RCC. Immunotherapy as a palliative consideration could show a better response and a modest change in overall survival [5,16].

## Conclusions

Metastatic RCC with sarcomatoid differentiation in the oral cavity is very rare. Sarcomatoid differentiation is associated with poor prognosis and unfavorable overall survival. When patients have multiple RCC metastases, surgery is not always the best treatment option, and palliative care is needed. In addition to other differential diagnoses, metastatic neoplasms should be considered when evaluating jaw cancers. It is important to consider the likelihood of metastatic illness, especially in individuals with a history of RCC. In the present case, the benign presentation of the lesion masqueraded as a possible malignant lesion, but with accurate clinical history and detailed histopathological examination, a conclusive diagnosis was obtained.
